# Causal relationship between sleep apnea and prostatitis: A 2-sample Mendelian randomization study

**DOI:** 10.1097/MD.0000000000040856

**Published:** 2024-12-13

**Authors:** Lifeng Chen, Jiaguo Huang, Ji Sun, Yi Fan, Yinfang Xie

**Affiliations:** a Department of Urology, Affiliated Xiaoshan Hospital, Hangzhou Normal University, Hangzhou, China.

**Keywords:** GWAS, Mendelian randomization, prostatitis, sleep apnea

## Abstract

Observational studies indicate that the risk of prostatitis in sleep apnea patients is higher than those without sleep apnea. However, the causal relationships remain to be determined. This study aims to investigate the causal relationships of sleep apnea on prostatitis using Mendelian randomization (MR). Summary-level data for sleep apnea (16,761 cases and 201,194 controls) and prostatitis (1859 cases and 72,799 controls) were available from the GWAS summary data. Two-sample MR analyses were performed to investigate the causal relationship between sleep apnea and prostatitis. The inverse variance weighted (IVW) analysis was employed as the primary statistical method. In 2-sample MR analyses, we found that IVW estimates revealed that sleep apnea inferred an effect on risk of prostatitis at statistical significance (odds ratio [OR] = 1.370, 95% confidence interval [CI] = 0.094–0.535, *P* = .005). This MR study strengthens the evidence of a causal relationship between sleep apnea and prostatitis in Europeans.

## 1. Introduction

Prostatitis is 1 of the major public health challenges in the management of urology, mainly caused by various factors such as bacterial infections and nonbacterial factors. The main clinical manifestations of prostatitis are urinary tract irritation symptoms and chronic pelvic pain. As the incidence of prostatitis has increased rapidly, it has greatly affected the patients’ quality of life.

International data indicates that only 5% to 10% of patients suffering from prostatitis have a microbiologically proven bacterial infection.^[1,2]^ The histologic studies have confirmed the presence of inflammatory changes in both bacterial and nonbacterial prostatitis. Most cases of chronic inflammation involve lymphocyte invasion, macrophage infiltration, and plasma cell infiltration.^[3,4]^ Smoking, alcohol consumption, elevated plasma glucose level, and lack of physical activity were linked to an increased risk of prostatitis-like symptoms.^[5]^

In a previous cross-sectional study involving 1236 sleep apnea patients and 4944 healthy controls, the risk of prostatitis was higher (odds ratio [OR] = 1.95, *P* < .001) than those without sleep apnea, and caused different levels of risk for multiple urological disorders.^[6]^ However, this study is a observational study and is more susceptible to confounding factors.

Compared to observational studies, Mendelian randomization (MR) is a novel study design based on genetic epidemiology which limits the bias caused by confounding and reverses causal relationships. In MR, genetic variants are utilized as instrumental variables (IVs) to deduce the causal relationship between the exposure and an outcome, which robustly related to a modifiable exposure.^[7,8]^ Owing to the application of genome-wide association study (GWAS), 2-sample MR has been extensively used in a variety of diseases. In this study, we attempt to investigate the causal relationship between sleep apnea and prostatitis using the 2-sample MR analysis based on the aggregated statistical data from large-scale GWAS.

## 2. Methods

Due to such a reanalysis of previously collected and published data, no additional ethics approval was needed.

**Figure 1.**
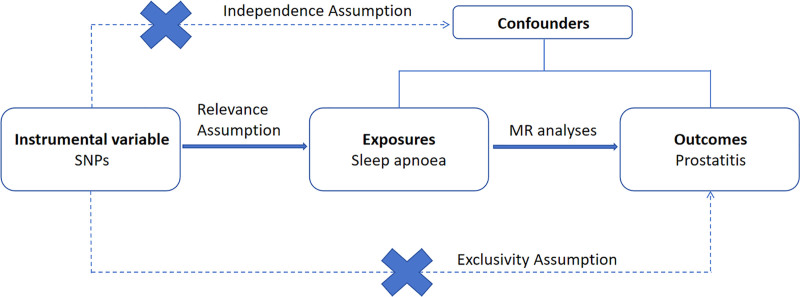
The overview of the study design.

In this study, the use of single nucleotide polymorphisms (SNPs) as IVs attempted to investigate the causal relationship between sleep apnea and prostatitis. The study design is illustrated in Figure 1. For this approach to work, 3 assumptions must be met: the SNPs must be strongly associated with sleep apnea; the SNPs should not affect confounding factors that may influence the relationship between exposure and outcome; and the SNPs ought to only influence the outcome through the exposure rather than any other pathways.

### 2.1. Genetic variants associated with sleep apnea and prostatitis

The study utilized genetic instruments to make an assessment on the causal relationship between sleep apnea and prostatitis. The primary genetic instruments were derived from the MR-base database.^[9]^ We extracted the SNPs associated with sleep apnea and prostatitis at genome-wide significance (*P* < 5 × 10^–6^) with the stringent pairwise linkage disequilibrium *r*^2^ < 0.001 from published GWAS meta-analysis (https://gwas.mrcieu.ac.uk/, search date: January 15, 2024). We selected SNPs for each exposure from the largest and most relevant GWAS of European ancestry participants. There were 16,761 cases and 201,194 control cases in the GWAS summary data for sleep apnea (GWAS ID: finn-b-G6_SLEEPAPNO). Totally, 217,955 cases were analyzed in the study. Additionally, there were 1859 cases and 72,799 control cases in GWAS summary data for prostatitis (GWAS ID: finn-b-N14_prostatitis). At the time of or after data collection, the authors did not obtain any information that identified individual participants. In order to determine whether these SNPs were related to any confounding factors (independence assumption), we checked them in the PhenoScanner V2 database of human genotype-phenotype associations (http://www.phenoscanner.medschl.cam.ac.uk/). Further, to evaluate their association with exposure, we assessed these IVs by *R*^2^ values and F statistic values.^[10]^ Following are the formulae, with the relevant variables noted.:


$$\begin{array}{l} R^{2}=2\times \left(1-MAF\right)\times \left(MAF\right)\times {\left(\frac{\beta }{SE\times \sqrt{N}}\right)}^{2} \end{array}$$

$$\begin{array}{l} F=\frac{N-k-1}{k}\;\;\;\times \;\;\;\frac{R^{2}}{1-R^{2}} \end{array}$$

Note: β, effect sizer; *k*, number of SNPs; MAF, minor allele frequency; N, sample size; SE, standard error.

### 2.2. Statistical analyses

All analyses were performed with the package TwoSampleMR (version 0.4.25) and MR-PRESSO (version 1.0) in *R* software (version 3.6.1). Before performing the MR analysis, we harmonized the effect alleles across the GWASs of sleep apnea and prostatitis. In order to determine the causal relationship between sleep apnea and prostatitis, we used the inverse variance weighted (IVW), weighted median, and MR-Egger to determine MR estimates of sleep apnea for prostatitis. Owing to different assumptions regarding horizontal pleiotropy, multiple approaches were used. The main analyses were conducted by the IVW, which is to evaluate the effect of each SNP on the outcome with the Wald ratio and to perform a meta-analysis for the combined causal effect. The main outcome, which can only be affected by instruments exposed to interest and not by alternatives^[11]^ To complement IVW estimates, MR-Egger and weighted median methods were used, which could provide more robust estimates but are less efficient (wider confidence interval [CI]). In this study, a tighten instrument *P* value threshold was imposed if these approaches produced inconsistent estimates.^[12]^

In MR studies, sensitivity analysis is crucial to detect underlying pleiotropy, and high heterogeneity can seriously impact MR estimates. Based on the IVW approach, potential horizontal pleiotropy was represented using heterogeneity markers (Cochran *Q*-derived *P* < .05). The intercept obtained from the MR-Egger regression with *P* < .05 to indicate the presence of directional pleiotropy that may be influencing the MR results.^[13]^ In addition, horizontal pleiotropy was evaluated and corrected through MR-Pleiotropy Residual Sum and Outlier methods (MR-PRESSO).^[12]^ MR-PRESSO consisted of 3 components: MR-PRESSO global test, MR-PRESSO outlier test, MR-PRESSO distortion test. With a lower percentage of horizontal pleiotropy variants than 10%, it has a less biased and has better precision than IVW and MR-Egger.^[14]^ To determine if MR estimates were influenced by a single SNP, a leave-one-out analysis was conducted.

## 3. Results

A total of 32 SNPs were recognized as being significantly associated with sleep apnea (*P* < 5 × 10^–6^, LD *r*^2^ < 0.001). Detailed information about these 32 SNPs can be found in Table S1, Supplemental Digital Content, http://links.lww.com/MD/O150. The F-statistics for the 32 SNPs, ranging from 20.845 to 66.584, exceed the conventional value of 10, suggesting a strong potential for these instruments. Using PhenoScanner V2 database of human genotype-phenotype association to ensure they were independent of any confounding factors (independence assumption), the following 24 independent SNPs (exposure data) and outcome data were harmonized to guarantee that the effect corresponded to the same allele (Table S2, Supplemental Digital Content, http://links.lww.com/MD/O150).

**Figure 2.**
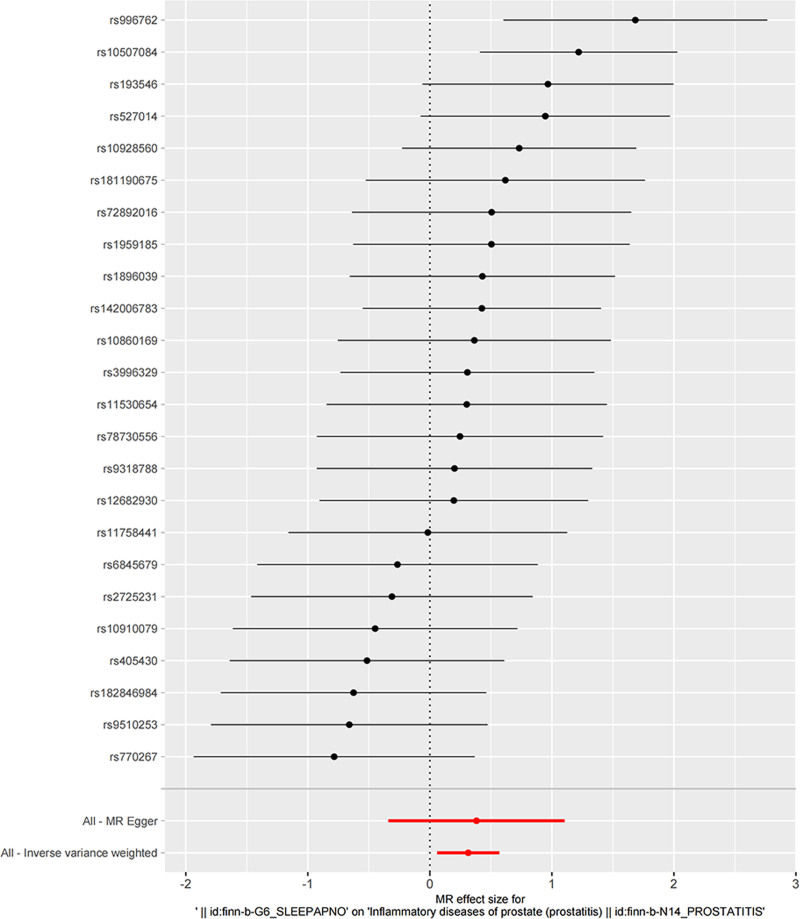
Forest plot of the causal effects of single nucleotide polymorphisms associated with sleep apnea and prostatitis.

**Figure 3.**
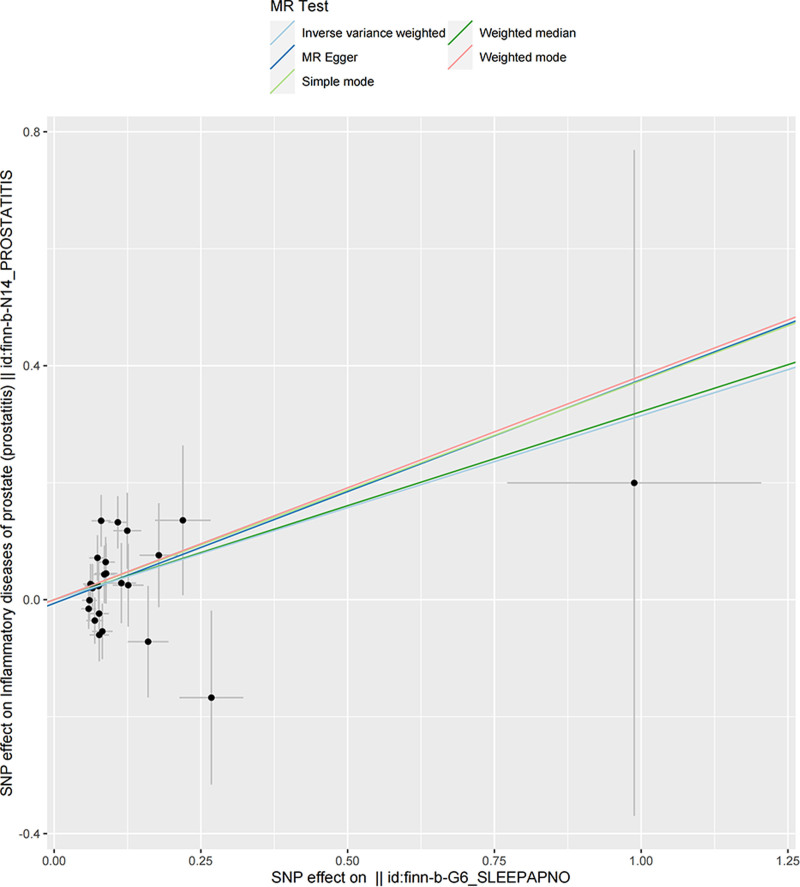
Scatter plot of the causal effects of single nucleotide polymorphisms associated with sleep apnea and prostatitis.

Before MR analyses were performed, heterogeneity was observed with a Cochran Q-test derived *P* value as .103 of MR-Egger and *P* value as .129 of IVW. Due to the lack of heterogeneity as indicated by Cochran Q-test, we used the fixed-effect IVW models in the main analysis. However, Moreover, MR-PRESSO analysis did not reveal any outliers. As shown in Figure 2, we found that IVW estimates revealed that sleep apnea inferred an effect on risk of prostatitis at statistical significance (OR = 1.370, 95% CI = 0.094–0.535, *P* = .005). While the direction of MR-Egger (OR = 1.465, 95% CI = −0.340 to 1.105), and the weighted median method (OR = 1.379, 95% CI = −0.003 to 0.646) estimates were consistent with IVW in Figure 3.

**Figure 4.**
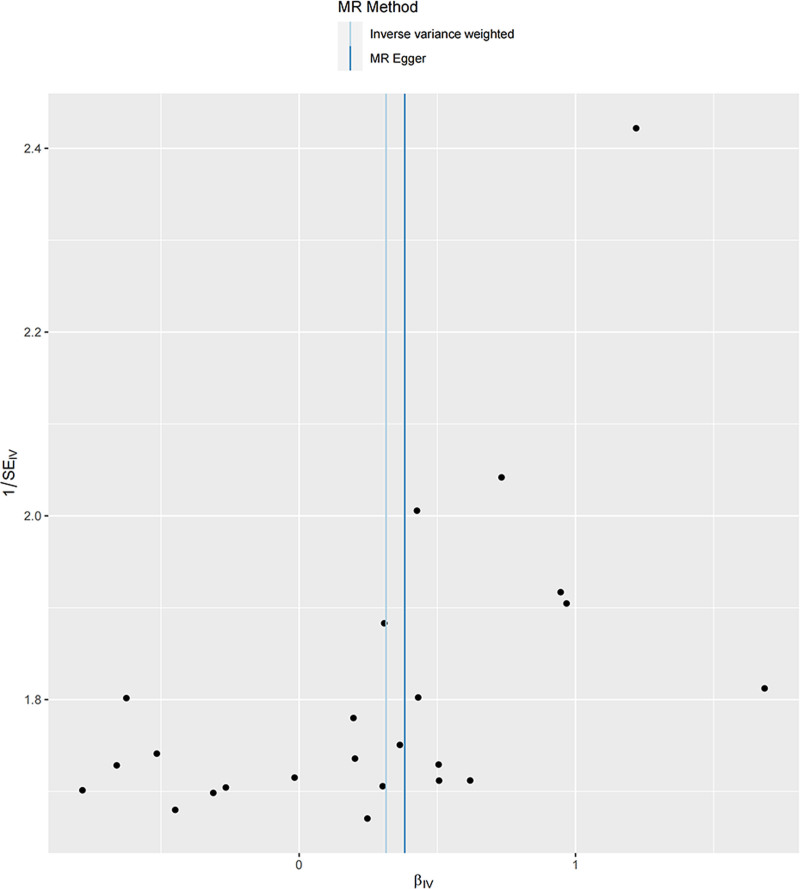
Funnel plot for the assessment of heterogeneity amongst single nucleotide polymorphisms associated with sleep apnea and prostatitis.

**Figure 5.**
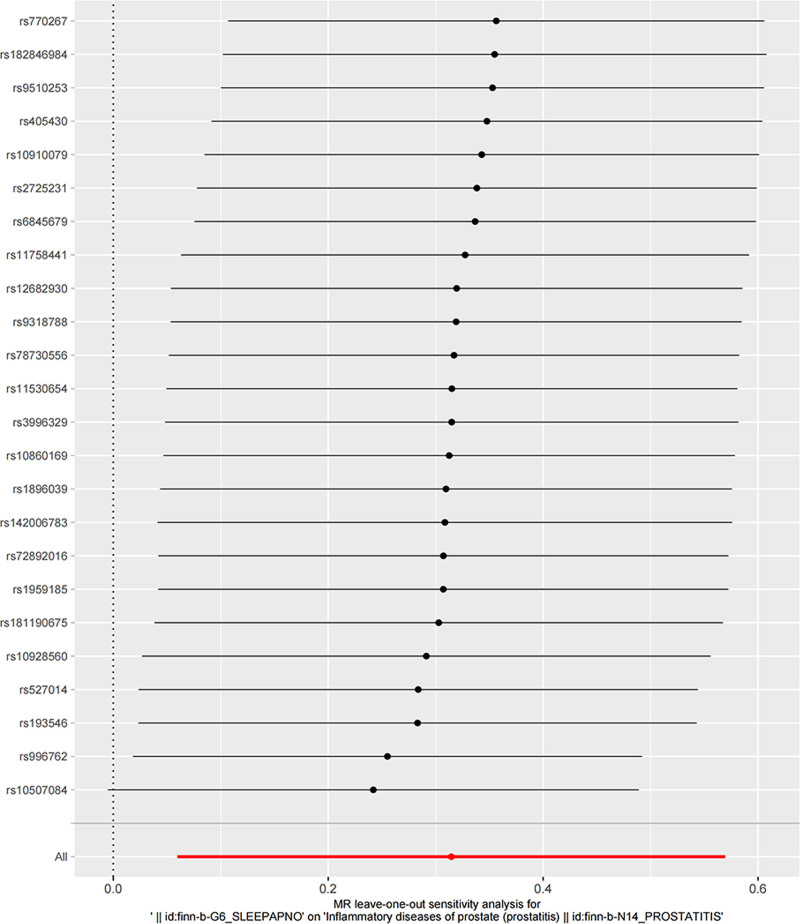
Leave-one-out of single nucleotide polymorphisms associated with sleep apnea and prostatitis.

Additionally, there was no evidence for a significant intercept (intercept = −0.001; SE = 0.010. *P* = .887), indicating that there was no directional pleiotropy observed. In the leave-one-out sensitivity analysis, no single SNP was intensely violated the overall effect of sleep apnea on prostatitis (Fig. 4). Additionally, as shown in Figure 5 the funnel plot showed symmetry, indicating that pleiotropy was not present.

## 4. Discussion

This study for the first time comprehensively explored sleep apnea in modifying the risk of prostatitis at the pathway, gene and SNP levels using GWAS data. Epidemiologic evidence suggests an association between sleep apnea and the risk of prostatitis.^[6]^ Most previous epidemiological studies have been case-control design and were difficult to clarify causal relationship by ambiguous chronological order. In addition, even in prospective observational studies, it is difficult to avoid the invasion of confounding risk factors. As a result of better study design in this study, we were able to confidently resolve causal relationships beyond bias using MR methods. We found that IVW estimates revealed that sleep apnea inferred an effect on risk of prostatitis at statistical significance (OR = 1.370, 95% CI = 0.094–0.535, *P *= .005). This result suggests a causal relationship between sleep apnea and prostatitis risk.

Sleep apnea is becoming an increasingly serious problem worldwide. As a sleep breathing disorder, sleep apnea is considered a significant risk factor for cardiovascular disease. It has been reported that sleep apnea is associated with systemic hypertension, ischemic heart disease, pulmonary hypertension and stroke.^[15]^ The characteristic of sleep apnea is repeated occurrence of the upper respiratory tract obstruction during sleep, followed by a decrease in blood hemoglobin oxygen saturation. Oxidative stress is initiated by repeated hypoxia and reoxygenation cycle, which are similar to hypoxia–reperfusion injury.^[16]^ Sleep apnea could induce excessive oxidative stress and systemic inflammation by tissue hypoxia.^[17]^ The cyclic pattern of intermittent hypoxia in sleep apnea triggers arterial chemoreceptors, enhancing the activity of sympathetic nervous system.^[18]^ This, in turn, influences the reactivity of vascular, promoting to the generation of free radicals. In oxidative stress, reactive oxygen species (ROS) are excessively formed due to an imbalance between oxidant-producing systems and antioxidant defence mechanisms. Aside from damaging various biomolecules and cellular components, ROS also act as important signaling molecules that inducte and activate multiple genes. ROS can oxidize proteins, lipids, carbohydrates and DNA, thereby altering their functions. A number of studies have shown that sleep apnea is related to increased levels of oxidative stress markers^[19–22]^ or decreased antioxidant defence.^[23]^ Oxidative stress is considered to be an important factor in the inflammatory cascade reaction of chronic prostatitis.^[24,25]^ In recent years, multiple studies have indicated oxidative stress plays a role in chronic prostatitis patients, regardless of the etiological basis.^[26,27]^ Therefore, sleep apnea can lead to oxidative stress and systemic inflammatory reactions, thereby increasing the risk of prostatitis.

A number of advantages can be gained from this MR research. Firstly, we can simulate randomized controlled trials in an observational setting by using an MR design. Randomized controlled traits are widely used and is largely well accepted when investigating causality, but are quite costly and often impractical. However, MR studies effectively mitigate the confounding bias associated with the random assignment of the SNP at conception. MR also avoids reverse causal effects compared to other observational studies. Secondly, our findings may influence the healthcare policy of sleep apnea versus prostatitis. In light of the high prevalence of sleep apnea and prostatitis in the general population, establishing a causal relationship between sleep apnea and prostatitis affects public health policies regarding early preventive intervention and timely treatment.

However, there are some limitations. Firstly, all GWAS data are obtained from European populations. Whether the findings of the studies we describe are consistent in other populations still remains to be investigated. Secondly, in order to conduct sensitivity and horizontal pleiotropy analyses, more SNPs had to be included as IVs, so instead of the traditional significance threshold (*P* < 5 × 10^–8^), we chose 5 × 10^–6^. Thirdly, the type, severity and course of prostatitis and the severity and course of sleep apnea were not described. And the sample size of prostatitis was still relatively small. A broader study containing a larger sample, subgroup analysis of prostatitis type and subgroup analysis according to severity could be considered in the future.

## 5. Conclusion

In conclusion, our MR analysis strengthens the evidence of a causal relationship between sleep apnea and prostatitis in Europeans. Even so, more studies are need to explore the potential biochemical mechanism and therapeutic targets for the preventive intervention of sleep apnea and prostatitis.

## Author contributions

**Conceptualization:** Jiaguo Huang, Yinfang Xie.

**Data curation:** Lifeng Chen.

**Formal analysis:** Lifeng Chen.

**Funding acquisition:** Jiaguo Huang.

**Methodology:** Yi Fan.

**Software:** Ji Sun.

**Supervision:** Yi Fan.

**Writing – original draft:** Lifeng Chen, Ji Sun.

**Writing – review & editing:** Jiaguo Huang, Yinfang Xie.

## Supplementary Material


