# Supplementary material for: Causal relationship between sleep apnea and prostatitis: A 2-sample Mendelian randomization study
Source: Medicine (Baltimore). 2024 Dec 13;103(50):e40856. doi: 10.1097/MD.0000000000040856 (PMC11651455; doi:10.1097/MD.0000000000040856)
Supplement: Supplementary file 1 [file medi-103-e40856-s001.docx]

Table S1. Single-Nucleotide Polymorphisms associated with sleep apnoea

| SNP | Effect allele | Other allele | EAF | BETA | SE | *P* | F |
| --- | --- | --- | --- | --- | --- | --- | --- |
| rs11587444 | G | A | 0.393 | 0.014 | 0.002 | 1.000×10-10 | 41.788 |
| rs11164870 | G | C | 0.605 | -0.012 | 0.002 | 4.200×10-08 | 30.037 |
| rs56188862 | C | T | 0.387 | -0.016 | 0.002 | 4.300×10-13 | 52.497 |
| rs1156588 | G | A | 0.210 | -0.015 | 0.003 | 2.900×10-09 | 35.241 |
| rs57462170 | A | G | 0.109 | 0.019 | 0.003 | 1.900×10-08 | 31.620 |
| rs2117137 | G | A | 0.405 | 0.013 | 0.002 | 1.700×10-09 | 36.338 |
| rs1481012 | G | A | 0.112 | -0.026 | 0.003 | 5.300×10-15 | 61.147 |
| rs34619 | A | G | 0.431 | 0.012 | 0.002 | 4.300×10-08 | 30.021 |
| rs72797284 | G | A | 0.271 | -0.017 | 0.002 | 7.000×10-13 | 51.558 |
| rs7757102 | G | A | 0.555 | -0.012 | 0.002 | 3.100×10-08 | 30.624 |
| rs2478875 | G | A | 0.209 | 0.022 | 0.003 | 5.100×10-17 | 70.299 |
| rs149805207 | G | A | 0.009 | -0.072 | 0.013 | 1.100×10-08 | 32.685 |
| rs4410790 | C | T | 0.631 | 0.041 | 0.002 | 3.400×10-76 | 341.268 |
| rs17685 | A | G | 0.278 | 0.023 | 0.002 | 1.600×10-22 | 95.364 |
| rs141071726 | A | G | 0.027 | 0.041 | 0.007 | 2.200×10-09 | 35.753 |
| rs9648476 | A | G | 0.623 | 0.013 | 0.002 | 1.100×10-08 | 32.722 |
| rs713598 | G | C | 0.402 | 0.013 | 0.002 | 5.200×10-10 | 38.590 |
| rs13282783 | T | C | 0.286 | -0.014 | 0.002 | 7.900×10-09 | 33.289 |
| rs56348300 | G | C | 0.185 | 0.016 | 0.003 | 6.100×10-09 | 33.799 |
| rs10764990 | A | G | 0.607 | -0.012 | 0.002 | 1.900×10-08 | 31.589 |
| rs10752269 | A | G | 0.506 | -0.013 | 0.002 | 1.300×10-09 | 36.878 |
| rs2351187 | A | G | 0.319 | 0.013 | 0.002 | 1.600×10-08 | 31.959 |
| rs17245213 | A | G | 0.208 | -0.015 | 0.003 | 2.000×10-08 | 31.521 |
| rs10741694 | C | T | 0.628 | 0.015 | 0.002 | 7.900×10-12 | 46.784 |
| rs1453548 | A | T | 0.665 | -0.013 | 0.002 | 3.000×10-09 | 35.167 |
| rs977474 | T | C | 0.834 | 0.022 | 0.003 | 2.400×10-14 | 58.180 |
| rs2783129 | G | C | 0.485 | -0.012 | 0.002 | 3.800×10-08 | 30.254 |
| rs17576658 | A | G | 0.247 | -0.013 | 0.002 | 4.100×10-08 | 30.116 |
| rs6829 | T | C | 0.596 | -0.012 | 0.002 | 3.700×10-08 | 30.282 |
| rs2645929 | G | A | 0.813 | -0.015 | 0.003 | 3.500×10-08 | 30.424 |
| rs12591786 | T | C | 0.159 | -0.018 | 0.003 | 3.700×10-10 | 39.274 |
| rs2472297 | T | C | 0.262 | 0.053 | 0.002 | 2.301×10-109 | 493.643 |
| rs9937354 | A | G | 0.424 | -0.014 | 0.002 | 4.900×10-11 | 43.231 |
| rs9302428 | G | C | 0.636 | 0.012 | 0.002 | 2.600×10-08 | 30.948 |
| rs2279844 | A | G | 0.379 | -0.012 | 0.002 | 4.000×10-08 | 30.151 |
| rs4808193 | C | T | 0.335 | 0.015 | 0.002 | 1.700×10-11 | 45.240 |
| rs57631352 | G | A | 0.297 | -0.013 | 0.002 | 1.700×10-08 | 31.868 |
| rs2273447 | T | A | 0.204 | 0.017 | 0.003 | 3.300×10-11 | 43.990 |
| rs4817505 | C | T | 0.390 | 0.015 | 0.002 | 4.200×10-12 | 48.012 |
| rs132904 | C | G | 0.779 | 0.017 | 0.003 | 7.800×10-11 | 42.296 |
| rs9624470 | A | G | 0.580 | 0.025 | 0.002 | 1.300×10-31 | 136.839 |

Note: SNP, single nucleotide polymorphism; REF, reference allele; EAF, effect allele frequency; beta, the effect size; SE, standard error

Table S2. Data for 24 SNPs as instrumental variables in exposure and outcome

| SNP | Effect allele | Other allele | EAF | BETA | SE | *P* | F |
| --- | --- | --- | --- | --- | --- | --- | --- |
| rs10910079 | T | C | 0.033 | 0.160 | 0.035 | 4.126×10-06 | 21.165 |
| rs527014 | T | C | 0.074 | 0.125 | 0.024 | 1.536×10-07 | 27.408 |
| rs996762 | G | C | 0.824 | -0.080 | 0.016 | 8.688×10-07 | 24.148 |
| rs10928560 | T | C | 0.195 | -0.088 | 0.016 | 2.802×10-08 | 30.879 |
| rs181190675 | T | C | 0.018 | -0.219 | 0.048 | 3.907×10-06 | 21.315 |
| rs182846984 | T | G | 0.014 | 0.268 | 0.054 | 8.360×10-07 | 24.251 |
| rs6845679 | T | C | 0.590 | 0.059 | 0.013 | 3.532×10-06 | 21.436 |
| rs2725231 | G | A | 0.834 | -0.077 | 0.017 | 3.995×10-06 | 21.293 |
| rs78730556 | T | C | 0.067 | -0.115 | 0.025 | 4.820×10-06 | 20.845 |
| rs11758441 | T | C | 0.378 | 0.060 | 0.013 | 2.978×10-06 | 21.777 |
| rs3996329 | A | G | 0.237 | -0.076 | 0.015 | 2.908×10-07 | 26.161 |
| rs405430 | T | G | 0.763 | -0.069 | 0.015 | 2.098×10-06 | 22.529 |
| rs12682930 | T | G | 0.061 | -0.126 | 0.026 | 1.494×10-06 | 23.201 |
| rs11530654 | C | A | 0.263 | 0.066 | 0.014 | 3.539×10-06 | 21.579 |
| rs10507084 | T | C | 0.179 | 0.109 | 0.016 | 2.797×10-11 | 44.307 |
| rs1896039 | A | G | 0.535 | 0.062 | 0.013 | 9.240×10-07 | 24.212 |
| rs10860169 | G | A | 0.291 | -0.065 | 0.014 | 2.012×10-06 | 22.718 |
| rs9318788 | G | A | 0.001 | 0.988 | 0.216 | 4.986×10-06 | 20.853 |
| rs9510253 | T | A | 0.147 | 0.082 | 0.018 | 3.082×10-06 | 21.760 |
| rs770267 | G | A | 0.831 | 0.077 | 0.017 | 3.834×10-06 | 21.460 |
| rs193546 | A | G | 0.744 | 0.074 | 0.014 | 2.266×10-07 | 26.706 |
| rs1959185 | A | G | 0.134 | 0.086 | 0.018 | 2.582×10-06 | 22.120 |
| rs142006783 | C | T | 0.038 | 0.178 | 0.033 | 4.813×10-08 | 29.730 |
| rs72892016 | A | G | 0.120 | -0.089 | 0.019 | 3.944×10-06 | 21.246 |

Note: SNP, single nucleotide polymorphism; REF, reference allele; EAF, effect allele frequency; beta, the effect size; SE, standard error
